# All trans retinoic acid alleviates coronary stenosis by regulating smooth muscle cell function in a mouse model of Kawasaki disease

**DOI:** 10.1038/s41598-021-93459-3

**Published:** 2021-07-05

**Authors:** Eisuke Suganuma, Satoshi Sato, Satoko Honda, Atsuko Nakazawa

**Affiliations:** 1 Division of Infectious Diseases and Immunology, Allergy, Saitama Children’s Medical Center, 1-2 Shintoshin Chuou-ku Saitama-shi, Saitama, 330-8777 Japan; 2 Division of Clinical Research, Saitama Children’s Medical Center, Saitama, Japan

**Keywords:** Cardiology, Medical research, Pathogenesis

## Abstract

Coronary artery (CA) stenosis is a detrimental and often life-threatening sequela in Kawasaki disease (KD) patients with coronary artery aneurysm (CAA). Therapeutic strategies for these patients have not yet been established. All-trans-retinoic acid (atRA) is a modulator of smooth muscle cell functions. The purpose of this study was to investigate the effect of atRA on CA stenosis in a mouse model of KD. *Lactobacillus casei* cell wall extract (LCWE) was intraperitoneally injected into 5-week-old male C57BL/6 J mice to induce CA stenosis. Two weeks later, the mice were orally administered atRA (30 mg/kg) 5 days per week for 14 weeks (LCWE + atRA group, n = 7). Mice in the untreated group (LCWE group, n = 6) received corn oil alone. Control mice were injected with phosphate-buffered saline (PBS, n = 5). Treatment with atRA significantly suppressed CA inflammation (19.3 ± 2.8 vs 4.4 ± 2.8, p < 0.0001) and reduced the incidence of CA stenosis (100% vs 18.5%, p < 0.05). In addition, atRA suppressed the migration of human coronary artery smooth muscle cells (HCASMCs) induced by platelet-derived growth factor subunit B homodimer (PDGF-BB). In conclusion, atRA dramatically alleviated CA stenosis by suppressing SMC migration. Therefore, it is expected to have clinical applications preventing CA stenosis in KD patients with CAA.

## Introduction

Kawasaki disease (KD) is an acute systemic vasculitis of unknown etiology that is mainly associated with coronary artery aneurysms (CAAs) and occurs primarily in young children^1,2^. A serious complication of CAA is CA stenosis, which includes thrombotic occlusion of CA in the acute phase and progressive CA narrowing due to intimal hypertrophy in the chronic phase. These pathological features of CA stenosis were actually identified in autopsy cases^3^. Recently, Fukazawa et al*.* performed a nationwide survey of KD patients with giant CAA in Japan. Myocardial infarction (MI) occurred in 18% of these patients, and severe cardiac events were likely to occur within 2 years of the onset of KD^4^. More recently, Miura et al*.* identified the risk factors for cardiac events in KD patients with CAA. Male sex and intravenous immunoglobulin (IVIG)-resistance were independent risk factors for acute coronary events^5^. Although these clinical studies revealed the natural history of KD patients with CAA, novel therapeutic strategies for these patients have not yet been investigated.

Recent clinical studies have reported useful therapeutic tools for anti-inflammatory agents such as IVIG^6^, prednisolone^7^, infliximab^8^ and ciclosporin^9^, especially during the acute phase. Therefore, according to the 25th Japanese nationwide Kawasaki disease survey, the incidence of cardiac sequelae was reduced to 2.6% in KD patients^10^. However, until now, due to the lack of a suitable animal model, no drug has been developed that is useful for improving the prognosis of KD patients who already have CAA. Pathological investigations of KD autopsy cases reported by Orenstein et al. demonstrated that luminal myofibroblastic proliferation (LMP) was associated with unique smooth muscle cell (SMC)-derived myofibroblasts that also caused progressive CA stenosis^11^. Of note, we discovered the characteristics of CA stenosis in the *Lactobacillus casei* cell wall extract (LCWE)-murine model, which was first reported by Lehman et al*.* in 1985 and 1988^12,13^. Histologically, this model is characterized by intimal hypertrophy due to SMC proliferation and migration following severe CA vasculitis, leading to CA stenosis^14^. Since the LCWE-induced vasculitis model closely resembled the pathological features found in human KD autopsy cases, we therefore decided to use this mouse as a model for chronic CA stenosis.

All-trans-retinoic acid (atRA) is a natural derivative of vitamin A that inhibits cell proliferation and migration and has anti-inflammatory properties^15–17^. Currently, atRA is a conventional therapy for the management of acute promyelocytic leukemia (APL)^18^. Recent experimental studies have reported that atRA treatment significantly reduces the formation of atherosclerosis in a high-fat diet-induced rabbit model^19^. Moreover, it has also been reported that atRA reduces neointimal formation in the carotid artery after balloon withdrawal injury in a rat model^20^. In addition, the synthetic retinoid Am80 significantly ameliorates *Candida albicans* water-soluble fraction (CAWS)-induced vasculitis through the inhibition of neutrophil migration and activation^21^. Based on previous animal studies, we hypothesized that atRA could suppress CA stenosis by modulating the properties of vascular smooth muscle cells (VSMCs) in a KD mouse model. In this study, we investigated the effect of atRA on LCWE-induced CA stenosis in a mouse model of KD.

## Results

### Preliminary evaluation of LCWE-induced CA stenosis

**Figure 1:**
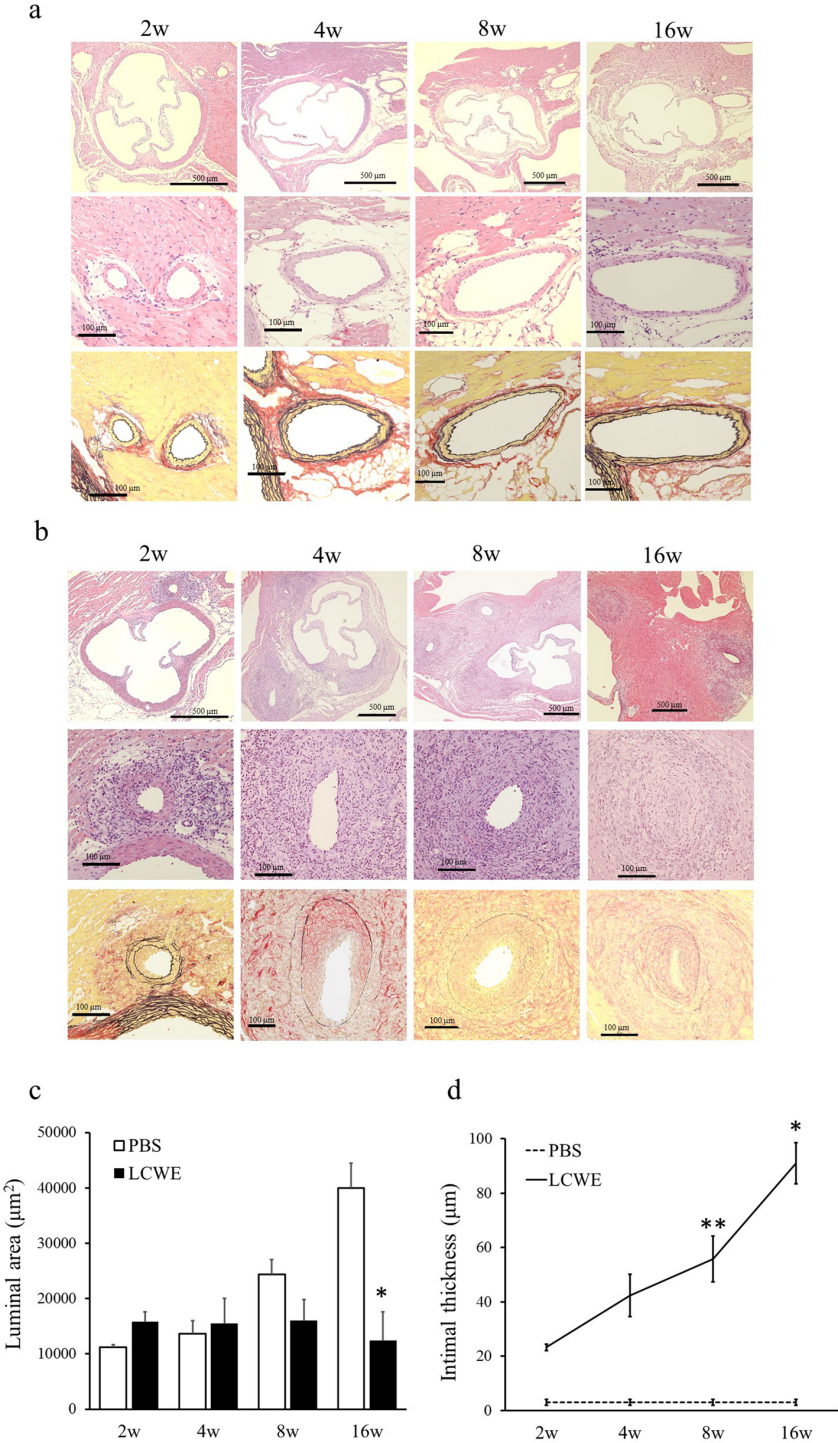
Natural history of LCWE-induced CA stenosis. Hematoxylin and eosin (H&E) and Elastica van Gieson (EVG) staining of aorta and CA sections from mice 2, 4, 8 and 16 weeks after PBS **(a)** and LCWE injection **(b)**. Time course of luminal area (μm^2^) **(c)**, and intimal thickness (μm) **(d)** after PBS (n = 3, each group) and LCWE injection (2w: n = 5, 4w: n = 6, 8w: n = 6, 16w: n = 7). The values are expressed as the mean ± SD. *p < 0.05 vs PBS (16w), **p < 0.0001 vs PBS (8w). All the data were generated from 1 experiment at the same timeframe.

The mice were sacrificed 2 (n = 5), 4 (n = 6), 8 (n = 6) and 16 weeks (n = 7) after LCWE injection to investigate the natural history of LCWE-induced CA stenosis. Elastica van Gieson (EVG) staining revealed that CA intimal formation was first observed at 2 weeks and that the intimal thickness gradually increased over time. In addition to the increased intimal thickness, maximum vessel luminal narrowing was observed at 16 weeks after LCWE administration (Fig. 1b). Therefore, we chose to begin atRA treatment 2 weeks after LCWE injection and continued treatment for the next 14 weeks, which coincided with the onset of intimal formation and the time of maximum CA stenosis in this mouse model (Fig. 1c,d). None of the control mice that were injected with phosphate-buffered saline (PBS) exhibited pathological changes (Fig. 1a) (n = 3 in each group).

### Effect of atRA on CA inflammation and stenosis

**Figure 2:**
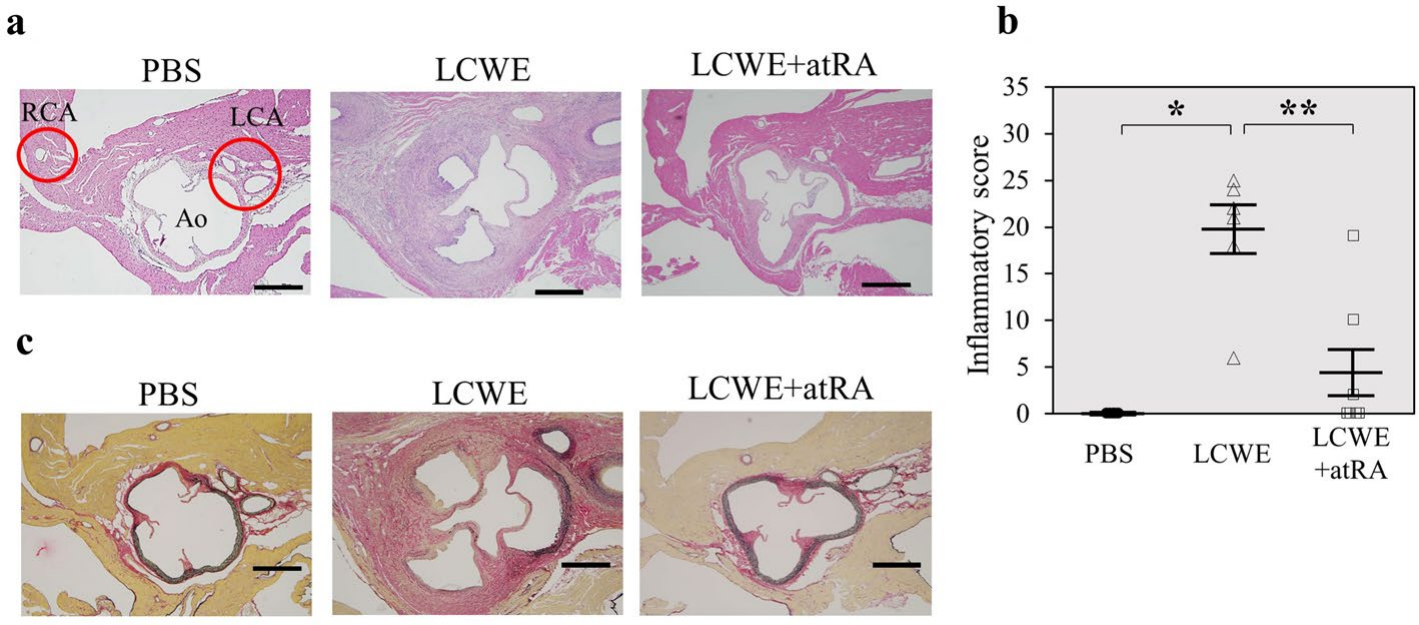
Effect of atRA on LCWE-induced vasculitis. Effect of atRA on LCWE-induced vasculitis. Mice were injected intraperitoneally with PBS (PBS, n = 5) or LCWE (n = 13). Two weeks later, LCWE-injected mice were further divided into two groups: no treatment (LCWE, n = 6) and atRA (LCWE + atRA, n = 7). All mice were sacrificed 16 weeks after LCWE or PBS injection. Representative photographs showing the cross-section of an aortic lesion containing the bilateral coronary arteries stained with H&E **(a)** and EVG **(c)**. RCA, right coronary artery; LCA, left coronary artery; Ao, aorta. Scale bar = 500 μm**. (b)** Semiquantitative evaluation of the CA inflammatory score in mice with PBS (n = 5)**,** LCWE (n = 6) and LCWE + atRA (n = 7). The values are expressed as the mean ± SD. *p < 0.05, **p < 0.0001. All the data were generated from 1 experiment at the same timeframe.

**Figure 3:**
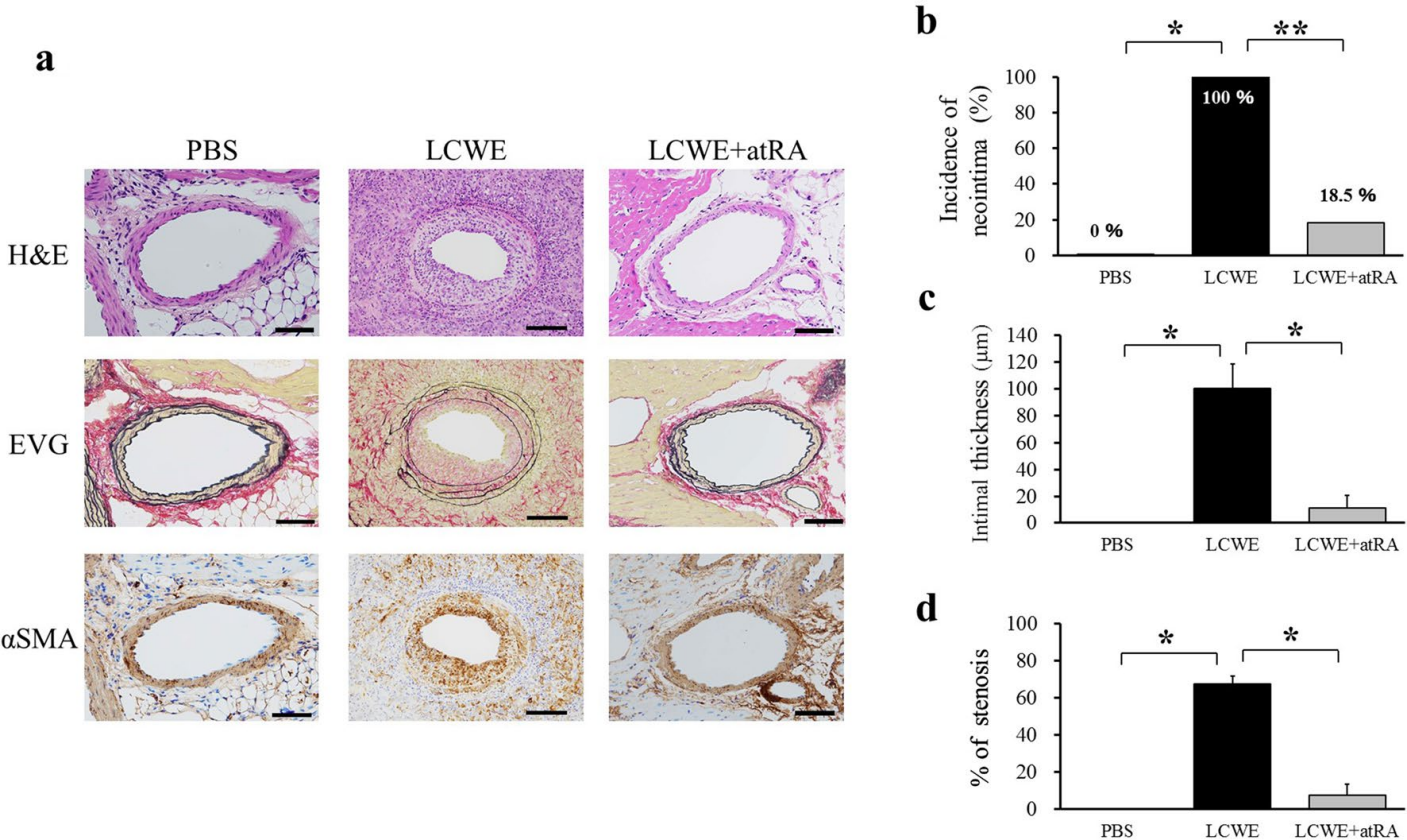
Effect of atRA on LCWE-induced CA stenosis. **(a)** H&E, EVG and α-smooth muscle actin (α-SMA) staining of CAs from mice treated with PBS (n = 5), LCWE (n = 6) and LCWE + atRA (n = 7)**.** Scale bar = 100 μm**.** The incidence of neointima **(b)**, intimal thickness **(c)** and % of stenosis **(d)** were compared among the groups. The data are expressed as the mean ± SD. *p < 0.01, **p < 0.05. All the data were generated from 1 experiment at the same timeframe.

**Figure 4:**
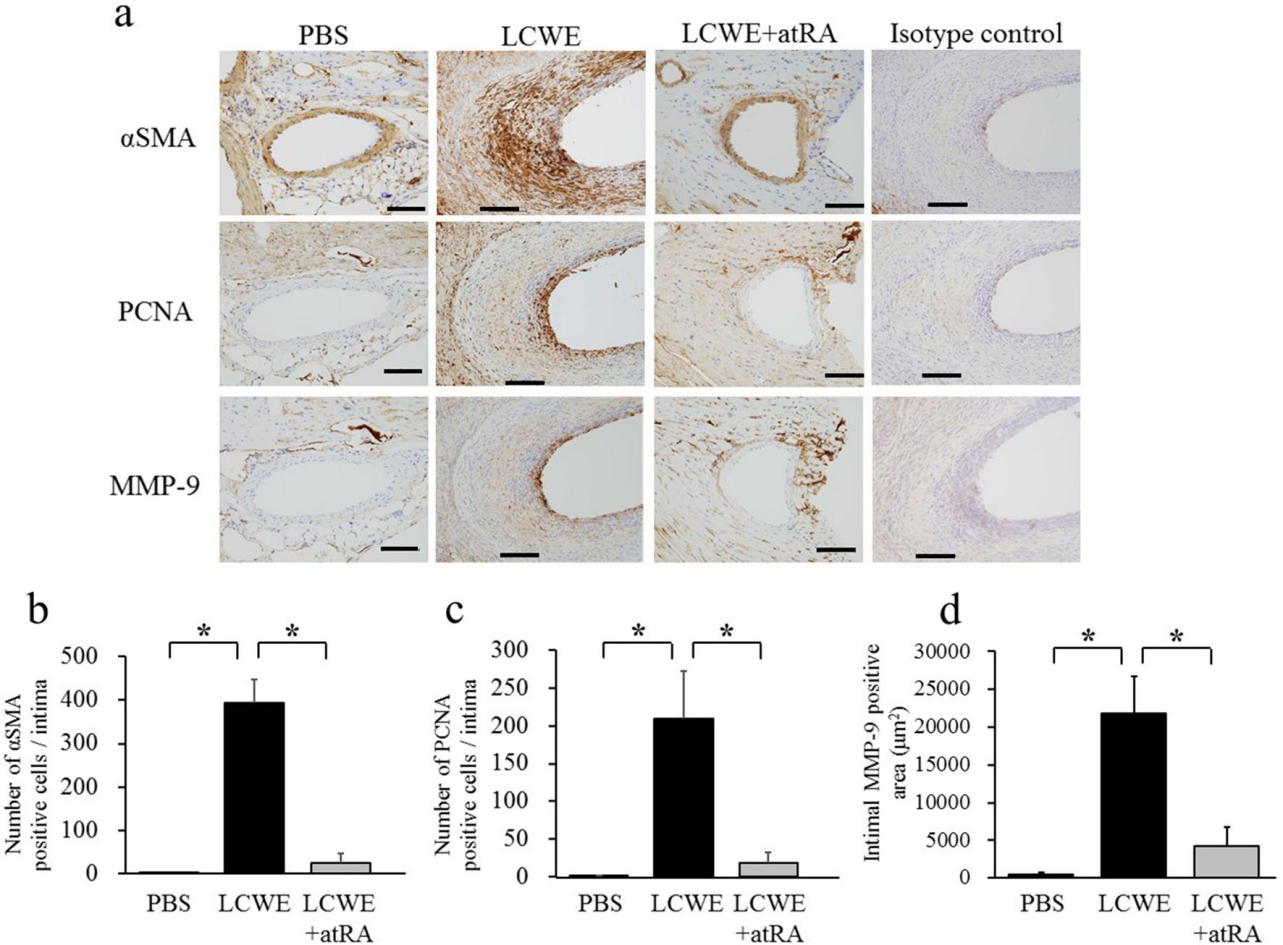
Immunohistochemical staining of α-SMA (upper), proliferating cell nuclear antigen (PCNA) (middle), and matrix metalloproteinase-9 (MMP-9) (bottom) in mice with PBS, LCWE and LCWE + atRA **(a)**. All positive cells are shown as a brown color. Isotype antibodies were used as negative control; α-SMA: mouse polyclonal IgG2a kappa, PCNA: rabbit IgG, MMP-9: mouse monoclonal IgG1a kappa. Scale bar = 100 μm. The number of intimal αSMA- **(b)** and PCNA-positive cells **(c)** and the intimal MMP-9-positive area **(d)** in mice treated with PBS (n = 5), LCWE (n = 6) and LCWE + atRA (n = 7) were quantitatively analyzed and compared among the groups. The data are expressed as the mean ± SD. *p < 0.0001. All the data were generated from 1 experiment at the same timeframe.

Next, we evaluated the effects of atRA on CA inflammation. atRA (20 mg/kg) was orally administered 5 days per week from 2 to 16 weeks after LCWE injection. Inflammatory cells predominantly infiltrated the aortic root, and bilateral CAs were observed in LCWE-induced mice compared to PBS-treated mice. atRA significantly suppressed CA inflammation (19.3 ± 2.8 vs 4.4 ± 2.8, p < 0.0001) (Fig. 2). Mice injected with LCWE exhibited CA stenosis in addition to vasculitis. We next assessed the effects of atRA on CA stenosis using three parameters. Representative microphotographs showing LCWE-induced CA intimal formation are shown in Fig. 3a. atRA significantly reduced intimal incidence (100% vs 18.5%, p < 0.05), intimal thickness (100.5 ± 18 vs 11.5 ± 9.3 μm, p < 0.01), and the % of CA stenosis (67.5 vs 7.6%, p < 0.01) (Fig. 3b–d). α-Smooth muscle actin (αSMA)-positive cells in the thickened intima of the CA were predominantly observed in mice injected with LCWE. Proliferating cell nuclear antigen (PCNA)-positive and matrix metalloproteinase (MMP)-9-positive cells were localized on the surface of the neointima but were not observed in mice treated with atRA. The isotype control for each staining revealed no evidence of positively stained cells or regions in cardiac sections from LCWE-injected mice (Fig. 4a). Figure 4 shows the quantification of the number of positively stained cells in the intima for αSMA and PCNA (Fig. 4b,c), and the positively stained area in the intima for MMP-9 (Fig. 4d). Compared with mice injected with PBS, LCWE-injected mice had significantly increased numbers of αSMA-positive cells (393 ± 53 vs 1.6 ± 0.4, p < 0.0001), PCNA (209 ± 63 vs 1.0 ± 0.8, p = 0.006) and MMP-9-positive area (21,775 ± 285 μm^2^ vs 465 ± 285 μm^2^, p = 0.002). However, the enhancement of αSMA, PCNA, and MMP-9 expression by LCWE was completely abolished by atRA administration.

### Effect of atRA on LCWE-induced elastin degradation through the suppression of MMP-9

**Figure 5:**
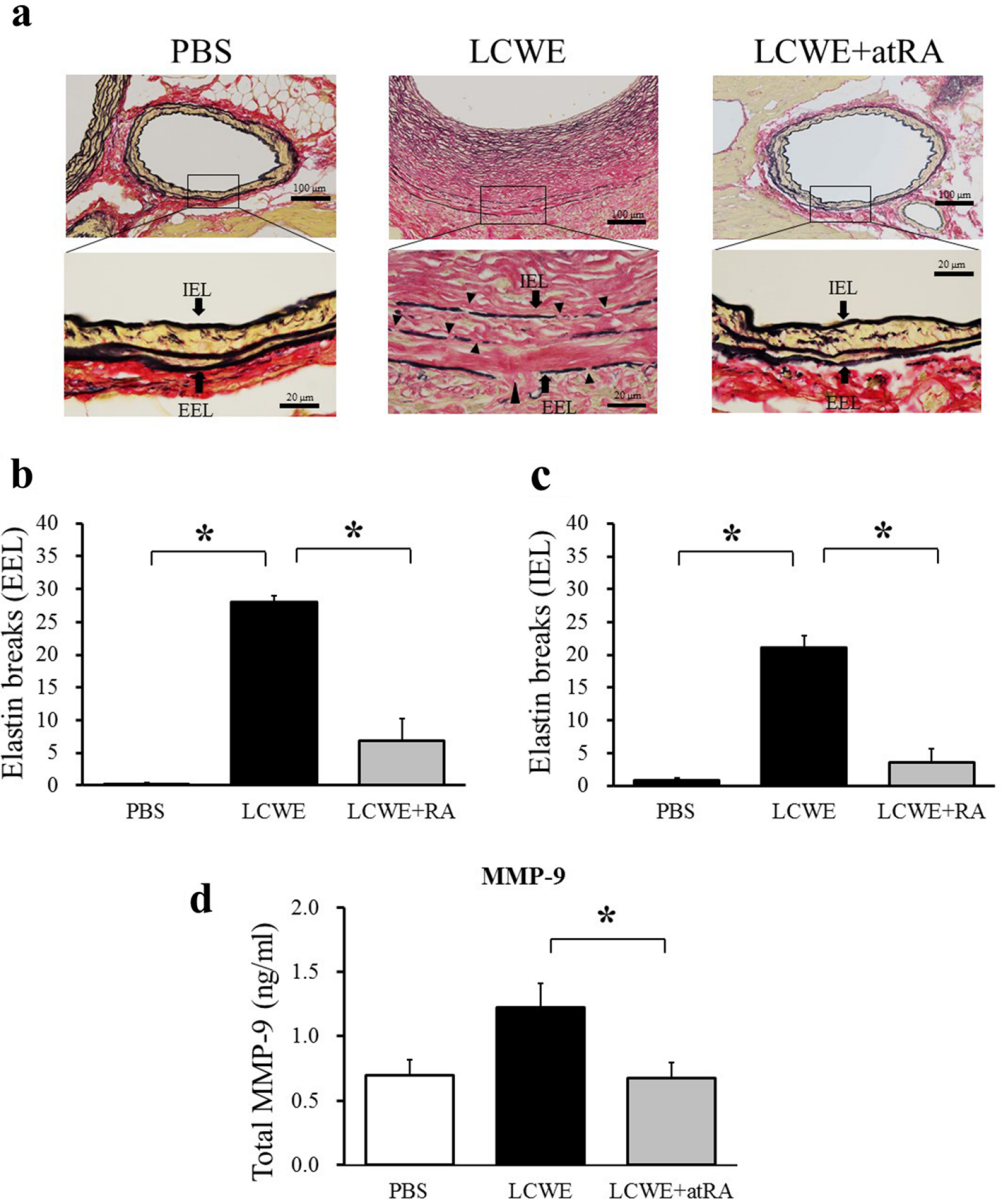
Effect of atRA on LCWE-induced elastin breaks. EVG staining of CAs from mice treated with PBS, LCWE and LCWE + atRA **(a)**. Elastin breaks in the external elastic lumina (EEL) **(b)** and in the internal elastic lumina (IEL) **(c)** among the groups (PBS: n = 5, LCWE: n = 6, LCWE + atRA: n = 7). **(d)** The serum level of MMP-9 in mice with PBS (n = 5), LCWE (n = 6) and LCWE + atRA (n = 7). The data are expressed as the mean ± SD. *p < 0.0001, **p < 0.05. All the data were generated from 1 experiment at the same timeframe.

Changes in the proliferative phenotype of SMCs precede elastolysis and are thought to play an important role in the development of intimal hyperplasia^22^. Therefore, assessing elastin degradation is extremely important for regulating intimal formation in the vessel wall. Next, we investigated the effect of atRA on the frequency of elastic breaks in the tunica media. LCWE-injected mice had more frequent interruptions and weakening of elastic fibers than mice that were administered PBS (Fig. 5a). atRA significantly reduced the elastin break scores of the external elastic lumina (EEL) (28 ± 1 vs 6.9 ± 3.4, p < 0.0001) and internal elastic lumina (IEL) (21.2 ± 1.7 vs 3.6 ± 2.1, p < 0.0001) (Fig. 5b,c). The potent electrolytic protein MMP-9, was increased in the serum of LCWE-induced mice (1.226 ± 0.18 ng/ml) compared with PBS-injected mice (0.697 ± 0.12 ng/ml, p = 0.66). This LCWE-induced increase in MMP-9 was significantly suppressed in atRA-treated mice (0.674 ± 0.12 ng/ml, p = 0.035 vs LCWE group) (Fig. 5d).

### Inhibitory effects of atRA on SMC migration and MMP-9 and TNF-α production in vitro

**Figure 6:**
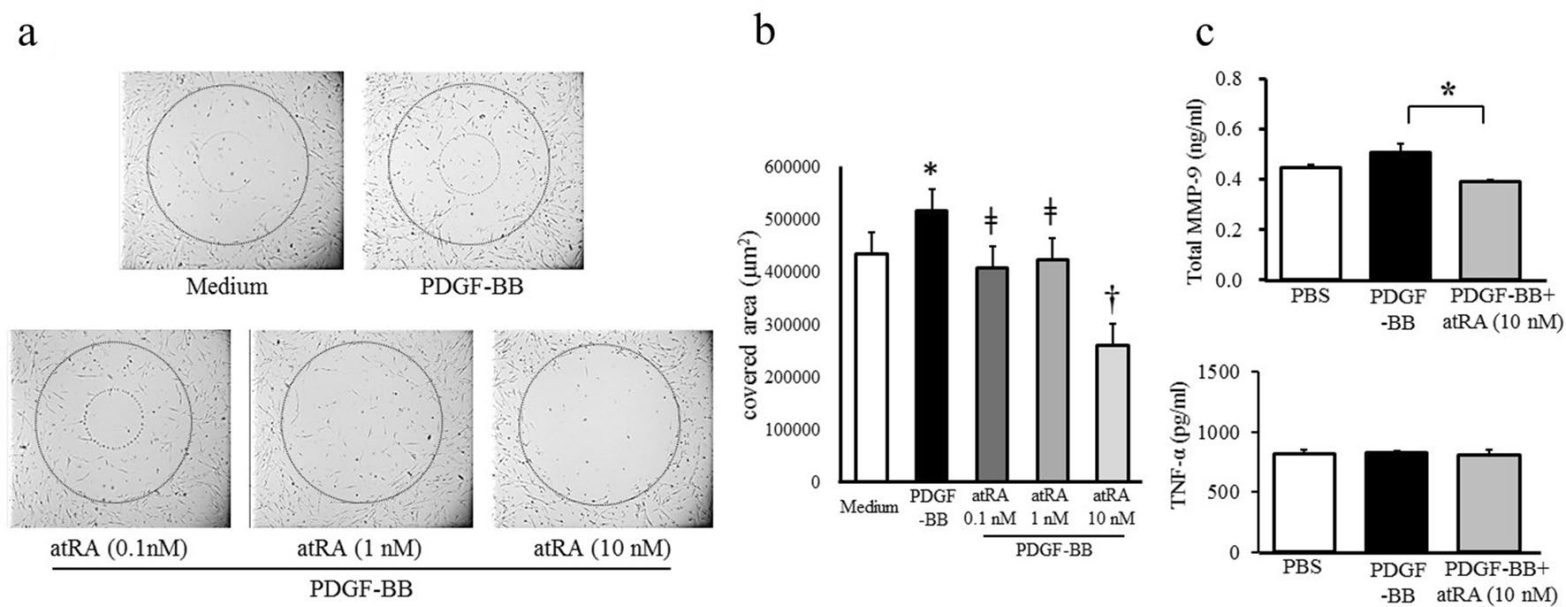
The migration of human coronary artery smooth muscle cells (HCASMCs). A total of 10,000 cells were incubated with PDGF-BB (20 ng/ml) to induce cell migration in the presence or absence of various concentrations of atRA (0.1, 1, and 10 nM) for 72 h **(a)**. Dotted circles indicate covered areas where the cells were not attached at baseline. The cells that migrated from the uncovered area (μm^2^) to the covered area were quantitatively measured in each group **(b)**. Magnification × 4. *p < 0.05 vs Medium, ǂp < 0.0001 vs PDGF-BB, atRA10 nM, †p < 0.01 vs PDGF-BB, Medium. HCASMCs (1 × 10^6^ cells) were placed in 60 mm culture dishes and incubated with PDGF-BB (20 ng/ml) in the presence or absence of 10 nM atRA for 24 h. Total MMP-9 activity and TNF-α concentration were measured in each group **(c)**. *p < 0.05. The values are expressed as the mean ± SD of quadruplicate determinations in 2 independent experiments.

Next, human coronary artery smooth muscle cells (HCASMCs) were used to investigate the effect of atRA on HCASMC migration (Fig. 6a). The cell migration assay revealed that platelet-derived growth factor subunit B homodimer (PDGF-BB) stimulation increased the area covered by migrated cells (n = 16, 515,703 μm^2^) compared to that of medium alone (n = 8, 443,594 μm^2^, p = 0.04). Cells were then treated with 0.1, 1.0, and 10 nM atRA for 72 h. The areas covered by migrated cells after treatment with 0.1 and 1 nM atRA were 407,610 (n = 16) and 424,162 μm^2^ (n = 16), respectively. While these concentrations of atRA induced significant reductions of 21% (p < 0.0001) and 18% (p = 0.002), respectively, compared to those in the PDGF-BB-treated group, a greater reduction of 49% was observed in the 10 nM atRA treatment group (n = 16, p < 0.0001 vs. PDGF-BB, 0.1, 1 nM atRA treatment) (Fig. 6b). To examine the direct effects of atRA on MMP-9 and TNF-α production in HCASMCs, ELISA analysis was conducted. Although total MMP-9 activity was not changed by the PDGF-BB group (0.51 ± 0.03 ng/ml) compared to the PBS group (0.45 ± 0.03 ng/ml, p = 0.145), atRA in addition to PDGF-BB significantly decreased total MMP-9 activity (0.39 ± 0.01 ng/ml, p = 0.003 vs PDGF-BB). There were no differences in TNF-α concentration among the groups (Fig. 6c).

## Discussion

In the present study, we found that atRA dramatically reduced intimal hyperplasia and alleviated CA stenosis in an LCWE-induced model of KD vasculitis. CA stenosis is clinically caused by thrombus formation or intimal hyperplasia and induces cardiac events such as cardiac ischemia, MI, and even sudden death^2,4^. The clinical definition of myocardial infarction (MI) denotes the presence of acute myocardial injury detected by abnormal cardiac biomarkers in the setting of evidence of acute myocardial ischemia. In addition, MI is defined pathologically as myocardial cell death due to prolonged ischemia^23^. Vascular smooth muscle proliferation plays a pivotal role in the development of intimal hypertrophy, causing CA stenosis in KD patients with CAA, as evidenced by autopsy studies^11^. Therefore, this is the first report focused on the prevention of CA stenosis by regulating the properties of SMCs in a mouse CA arteritis model.

atRA is the most active metabolite of vitamin A. Numerous studies have reported that atRA has biological effects on various types of tumors, including breast and lung cancer and APL^24^. In recent years, atRA has been used as the standard therapeutic drug for the treatment of adult APL and pediatric neuroblastoma^25^. On the other hand, several basic experimental studies of cardiovascular disorders have shown that atRA has antiproliferative and antimigratory effects in animal models of intimal hyperplasia. Miano et al*.* showed that atRA reduced neointimal formation and promoted favorable geometric remodeling of the rat carotid artery after balloon withdrawal injury^20^. In addition, Zhang et al*.* showed that atRA suppressed neointimal hyperplasia and inhibited VSMC proliferation and migration through direct activation of AMP-activated protein kinase (AMPK) and inhibition of mTOR signaling^26^. Therefore, we hypothesized that atRA might exert beneficial effects on the CA stenosis mouse model we developed in recent years. These previous data indicated that atRA improved intimal proliferation mainly associated with αSMA-positive cells. However, the effectiveness of atRA on cardiovascular disorders in clinical practice has not yet been verified. In addition, several clinical studies have investigated the relationship between retinol binding protein 4 (RBP4) and KD. Kimura et al*.* showed that RBP4, which is a candidate diagnostic marker, was decreased in patients with acute KD^27^. Recently, Yang et al*.* reported that KD patients had significantly lower RBP4 levels than healthy controls, suggesting that RBP4, which is a main retinol transport protein, is closely associated with markers of inflammation and thrombogenesis in children with KD^28,29^.

Notably, compared to untreated mice, mice treated with atRA had significantly reduced CA inflammatory scores. This anti-inflammatory effect was consistent with the data reported by Miyabe et al*.* Am80, a retinoic acid receptor (RAR) agonist, has been shown to ameliorate mouse vasculitis induced by CAWS by suppressing neutrophil migration and activation^21^. In our study, the underlying pathophysiological mechanism of the anti-inflammatory effect of atRA remains unclear, but it is hypothesized that the SMC phenotype predisposes patients to increased proliferation and migration and contributes to persistent inflammation of the vessel wall. VSMC phenotypic switching is characterized by loss of a contractile phenotype that lacks VSMC markers including αSMA, calponin and SM22/tagln and acquires increased capacity for cell proliferation, migration and secretion of various extracellular matrix proteins and cytokines^30^. In contrast, it was unexpected that an increase in αSMA-positive cells in the CA intima was observed in mice injected with LCWE. However, this phenotypic switch is considered the hallmark of vascular repair, a reversible process wherein VSMCs return to their contractile state after completing vessel repair^31^. This may be a possible reason why abundant αSMA-positive cells were observed at the site of CA stenosis in LCWE-injected mice.

We found that atRA significantly decreased elastin breaks and suppressed serum MMP-9 activity. A previous study by Axel et al*.* revealed that atRA inhibited human SMC proliferation and significantly inhibited the protein expression and activity of MMP-2 and MMP-9 in vitro^32^. More recently, Xiao et al*.* reported that atRA attenuated the progression of angiotensin II-induced abdominal aortic aneurysms by downregulating MMP-2 and MMP-9 expression in abdominal aortic tissue in apolipoprotein E-knockout mice^33^. In addition, Bunton et al*.* reported that phenotypic alterations in VSMCs preceded elastolysis in a mouse model of Marfan syndrome^22^. Therefore, it is reasonable to hypothesized that atRA protects against elastin degradation through the downregulation of MMP-9 activity, which in turn results in the suppression of proliferative phenotypic switching and the inhibition of intimal hyperplasia.

Moreover, there was no significant increase in serum MMP-9 levels in LCWE-injected mice compared to PBS-infected mice. However, cardiac sections of LCWE-infected mice showed that local expression of MMP-9 was clearly enhanced. This is a noteworthy finding, and we believe that higher expression of MMP-9 predisposes patients to facilitate elastin breakdown and cause CA stenosis. Therefore, it may be hypothesized that serum MMP-9 had already peaked at the time of sacrifice, but local MMP-9 expression remained.

In vitro, we showed direct inhibitory effects of atRA on migration using HCASMCs stimulated with PDGF-BB. Several in vivo and in vitro studies have investigated the pathways that regulate the migration or proliferation of VSMCs. Day et al*.* first reported that atRA inhibited airway SMC migration by modulating the phosphatidylinositol 3 kinase (PI3K)/Akt pathway^34^. In addition, Zhang et al*.* demonstrated that atRA might inhibit neointimal hyperplasia and suppress VSMC proliferation and migration by direct activation of AMP-activated protein kinase (AMPK). They concluded that AMPK might be the pharmacological target of ATRA and that activation of AMPK by atRA may be a novel treatment strategy for atherosclerosis^26^. More recently, Yu et al*.* reported that atRA prevented vein graft stenosis by inhibiting Rb-E2F-mediated cell cycle progression in human vein SMCs^35^. It was also reported that the Rb-E2F pathway was required for PDGF-BB-induced VSMC proliferation^36^. Our results confirmed that atRA inhibited PDGF-BB-induced HCASMC migration, suggesting an association between the Rb-E2F pathway and the antiproliferative effect of atRA. Our research has some limitations. First, it was not possible to clearly determine whether atRA had a significant effect on inflammatory suppression or the induction of vascular repair. This is because pathological evaluations at different time points and cytokine/chemokine measurements during the experimental periods were not performed. One possibility remains that atRA provides complete protection from the development of CA inflammation and stenosis during the experimental period. Treatment with atRA was started relatively early in this mouse model. Therefore, it was considered necessary to select a schedule for later administration of atRA treatment when CA inflammation had already developed. Second, the therapeutic goal of KD patients with CAA is to not only promote the regression of CA aneurysms but also prevent further cardiac events such as acute MI and sudden death. Thus, the beneficial effect of atRA on the suppression of intimal hyperplasia may lead to protection against CA stenosis and these harmful events. On the other hand, excessive inhibition of intimal hyperplasia may delay aneurysmal regression, resulting in a residual aneurysm. Therefore, it is important to induce favorable SMC proliferation rather than completely suppressing intimal hyperplasia leading to CA stenosis. Additional research is needed to elucidate the mechanism of the beneficial effects of atRA. Third, no experiments were conducted in this study to investigate the distribution or type of retinoic acid receptors that could be expressed at the site of coronary arteritis. Moreover, we did not examine any specific signaling for retinoic acid receptors that could be measured in CA. Further research will be needed to clarify the mechanism by which atRA directly or indirectly influences vascular inflammation and SMC phenotypic switching via the retinoic acid signaling pathway. Fourth, it was not clear whether retinoic acid inhibits vascular stenosis by suppressing immune cell infiltration or directly regulating the SMC phenotype. We assumed that both of these potential mechanisms were involved in this beneficial effect of atRA. An In vitro study reported by Xu et at. reported that atRA inhibited lipopolysaccharide-induced proinflammatory cytokines (TNF-α, IL-1β and IL-6)^37^. Most of these cytokines are closely associated with the pathophysiology of Kawasaki vasculitis. Furthermore, it should be emphasized that both IL-1α and IL-1β are directly associated with the LCWE-induced KD mouse model of vasculitis^38,39^. In addition, Fujiu et al. showed that the synthetic retinoid Am80 suppresses smooth muscle phenotypic modulation and in-stent neointima formation by inhibiting KLF5^40^. Therefore, additional in vivo and in vitro experiments will be needed in the future to more clearly distinguish the main benefits of atRA for anti-inflammatory effects or regulating the SMC phenotype. Finally, for clinical application, Miyabe et al*.* reported that a synthetic retinoid, Am80 significantly suppressed CAWS-induced vasculitis via regulation of neutrophil migration and activation, suggesting that Am80 was expected to be useful in the acute phase of KD vasculitis in this study^21^. On the other hand, our research focuses on the ability of atRA to regulate SMC function, including the regulation of SMC migration and MMP-9 activity associated with CA stenosis. Compared to Miyabe’s study, our study provided a different mechanism of atRA that is beneficial to patients with CA aneurysms in terms of preventing cardiac events such as angina pectoris and MI. This is a new discovery in our current experiment.

In conclusion, atRA dramatically reduced CA inflammation and stenosis by suppressing the production of MMP-9 and the migratory properties of SMCs by regulating cellular functions. Therefore, atRA, which has both anti-inflammatory effects and the ability to repair the vascular wall, is expected to have clinical applications to prevent CA stenosis in KD patients with CAA.

## Methods

### Experimental protocol

Five-week-old male C57BL/6 J mice were purchased from CLEA Japan (Tokyo, Japan) and maintained in an environment with a 12 h light/12 h dark cycle under specific pathogen-free conditions. First, we examined the natural course of coronary stenosis in mice after LCWE administration. The mice were sacrificed on weeks 2, 4, 8, and 16 after intraperitoneal injection of 1000 µg of LCWE (n = 5 to 7 in each group). Control mice received PBS (n = 3 in each group). Cardiac tissue was harvested, and histological assessments were performed in detail as follows. Based on the pathological features of CA stenosis observed in the preliminary natural course experiment, the effects of atRA on CA stenosis in this mouse model of KD were examined. Five-week-old male C57BL/6 J mice (n = 13) were intraperitoneally injected with 1000 μg of LCWE. Two weeks later, these mice were divided into two groups. Mice were orally administered atRA (Sigma-Aldrich, St. Louis, MO, USA) at a dose of 20 mg/kg (n = 7) or an equivalent volume (0.2 ml) of corn oil (n = 6) using a disposable flexible-type gastric tube (Fuchigami, Kyoto, Japan) 5 days per week for 16 weeks. The same dose of atRA has been shown previously to inhibit neointimal hyperplasia and suppress the proliferation and migration of VSMCs in mice^26^. Control mice (n = 5) were injected with PBS instead of LCWE. All mice were killed at 18 weeks after LCWE or PBS administration. All mouse experimental procedures were performed in accordance with institutional guidelines and regulations of Saitama Children’s Medical Center, Japan. This animal experiment was performed with the approval of the Animal Experimental Ethics Committee of Saitama Children’s Medical Center (No: 2020-003). All experiments were performed in accordance with relevant ARRIVE guidelines.

### LCWE preparation

LCWE was prepared as previously described^14,41^. Briefly, *Lactobacillus Paracasei subsp. paracasei* (ATCC 11578; American Type Culture Collection, Manassas, VA, USA) was cultured in MRS broth (BD Difco, Franklin Lakes, NJ, USA) for 48 h at 37 °C. The cells were harvested and washed with PBS, after which the cells were disrupted in 2 packed volumes of 4% sodium dodecyl sulfate (SDS) overnight at room temperature. Cell wall fragments were extensively washed 10 times with PBS to remove any residual SDS. The SDS-treated cell wall fragments were sonicated (5 g of packed wet weight in 15 ml of PBS) for 2 h using a Q500 sonicator with a ¾” diameter probe at an amplitude setting of 70% (QSonica LLC, CT, USA). During sonication, the cell wall fragments were maintained by cooling in a dry ice/ethanol bath. The supernatant was centrifuged for 1 h at 20,000*g* at 4 °C, and the supernatant containing the cell wall extract was used for the experiments. The concentration of LCWE was determined based on the rhamnose content as measured by a phenol–sulfuric acid colorimetric assay and adjusted to 5000 µg/ml. To induce coronary arteritis/stenosis in mice, 1000 µg (0.2 ml) of the LCWE preparation was injected intraperitoneally.

### Histological evaluation

As a preliminary natural course experiment, we measured the luminal area and intimal thickness of the CA artery at 2, 4, 8, and 16 weeks after administration of PBS or LCWE. The luminal area was measured in a total of 6 coronary arteries per animal and expressed as the average luminal area (μm^2^) for each CA. Intimal formation was defined as the thickened intima afferently overhanging the inside of the internal elastic lamina (IEL). We chose to use intimal thickness as an indicator of intimal formation. The length of the intima at four sites was measured from CA and averaged, followed by counting 6 coronary arteries for each animal and representing the average values of the thickened intima of each CA.

An inflammatory assessment of the CAs was performed as described previously^14^. Briefly, 2.5-μm sections of cardiac tissue were stained with hematoxylin and eosin (H&E) and EVG. Then, we selected 5 consecutive sections slightly distal to the bifurcation of the bilateral CAs. The intensity of coronary arteritis was scored with the following four criteria: 0, no inflammation; 1, inflammatory cells in only the adventitia; 2, inflammatory cells in both the intima and adventitia; and 3, panvasculitis as previously described^42^. Ultimately, the inflammatory score is expressed as the total score of 10 CAs per individual animal. For the quantitative parameters of CA stenosis, we evaluated the incidence of neointima, intimal thickness and % of CA stenosis. The incidence of neointima implied the frequencies of individuals with intimal thickening of the CA and expressed as percentages in each group. Intimal thickness was previously described as above. % of CA stenosis was calculated from the formula [all regions within IEL − Remaining lumen region/All regions within IEL × 100 (%)].

Elastin breaks were scored on the following scale: score 0, no interruption of elastic fibers; score 1, elastic breaks ≤ 10; 2, elastic breaks ≻10; and score 3, obscuration or disappearance of elastic fibers. Elastin breaks were defined as interruptions in the elastin fiber and the reappearance of the fiber and are expressed as the total number of breaks in five consecutive sections. The IEL and EEL of the bilateral CAs were evaluated. In addition, coronary stenosis was assessed according to three different parameters, including the incidence of neointimal formation, intimal thickness (μm), and % of CA stenosis, which were measured with an NIS-Elements AR Ver5.11.00 (Nikon Instruments, Inc, Tokyo, Japan).

### Immunohistochemistry

Paraffinized tissue sections (2.5 μm-thick) from experimental mice were stained with the following antibodies: anti-α-SMA (1:1000, SMA clone 1A4, DAKO), anti-PCNA (1:800, anti-PCNA antibody, Abcam) and anti-MMP-9 (1:400, MMP-9, Santa Cruz Biotechnology, Inc.). In each staining group, isotype controls without the primary antibody were included and exhibited no staining. In addition, αSMA and PCNA quantification was performed by counting positively stained cells in the intima and was expressed as the number of positive cells/high power field (HFP) in the intima. For the quantification of MMP-9, histological software (NIS-Elements AR ver5. 11.00) was used to measure the intimal MMP-9-positive area (μm^2^)/intima.

### Cell culture and migration assay

Primary HCASMCs were purchased from Thermo Fisher Scientific K.K. (Tokyo, Japan) These cells were cultured in Dulbecco’s modified Eagle’s medium (3.15 g/L glucose and 15 mM l-glutamine DMEM) (Lonza, Tokyo, Japan) containing 10% fetal bovine serum (FBS) and a penicillin–streptomycin-amphotericin B mixture (Lonza, Tokyo, Japan) at 37 °C in a humidified atmosphere containing 5% CO_2_. The culture medium was changed every 24 h. Once the HCASMCs had grown to approximately 80 to 90% confluence, the cells were detached with a trypsin EDTA solution (Thermo Fisher Scientific, Tokyo, Japan). HCASMC migration assays were performed using an Oris cell migration assay kit (Platypus Technologies, LCC, WI, USA) according to the manufacturer's instructions. Briefly, cells were seeded at 10,000 cells/well in 96-well plates, and each well was coated with collagen I with a stopper in the central area to prevent the cells from adhering to the detection zone; the cells were incubated in serum-free medium for 24 h. Immediately after the stopper was removed, the cells were cultured with 20 ng/ml PDGF-BB in the absence or presence of various concentrations of atRA (0.1, 1.0, and 10 nM). After 72 h of incubation, cells that migrated from the perimeter to the detection zone were measured using an IN Cell Analyzer 2200 (Cytiba, MA, USA). The number of migrated cells in each well was counted in low-power (4 ×) fields.

### Measurement of total MMP-9 activity, TNF-α

Serum-starved HCASMCs (1 × 10^6^ cells) were placed in 60 mm culture dishes and incubated for 24 h at 37 °C. The cells were then incubated with medium alone, PDGF-BB (20 ng/ml) without atRA or in the presence of atRA at 10 nM for the next 24 h. Cell culture supernatants were collected and total MMP-9 activity (MMP-9 activity assay kit for humans, Cosmo Bio Co., Ltd, Tokyo, Japan) and TNF-α concentration (Human TNF-α immunoassay, R&D Systems, Inc. MN, USA) were measured according to the manufacturer's instructions. The data are expressed as ng/ml for MMP-9 and pg/ml for TNF-α.

### Measurement of total MMP-9 activity in mouse serum

Blood samples were extracted directly from the left ventricle using a 1 ml syringe with a 27-gauge needle immediately before sacrifice under deep anesthesia. The blood samples were centrifuged at 1500 rpm at room temperature and stored at − 80 °C until use. The serum MMP-9 level was measured by an MMP-9 activity assay kit for mice (Cosmo Bio Co., Ltd, Tokyo, Japan) according to the manufacturer’s instructions, and the data are expressed as ng/ml.

### Statistical analysis

All values are presented as the mean ± standard error of the mean (SEM). Statistically significant differences between mean values were determined using a two-tailed Mann–Whitney test. Statistical differences among the three or more groups were determined by one-way ANOVA followed by Bonferroni post hoc test. Differences in which p < 0.05 were considered statistically significant. IBM SPSS Statistics for Windows Version 24.0 (SPSS Japan, Tokyo, Japan) was used to analyze the data.

## Data Availability

All data generated or analyzed during this study are included in this published article.
